# Heme Oxygenases: Cellular Multifunctional and Protective Molecules against UV-Induced Oxidative Stress

**DOI:** 10.1155/2019/5416728

**Published:** 2019-11-21

**Authors:** ShiDa Chen, XiaoYu Wang, Muhammad Farrukh Nisar, Mao Lin, Julia Li Zhong

**Affiliations:** ^1^The Base of “111 Project” for Biomechanics & Tissue Repair Engineering; Key Laboratory of Biorheological Science and Technology, Ministry of Education, Bioengineering College, Chongqing University, Chongqing 400044, China; ^2^Chongqing Traditional Chinese Medicine Hospital, Chongqing 400011, China

## Abstract

Ultraviolet (UV) irradiation can be considered as a double-edged sword: not only is it a crucial environmental factor that can cause skin-related disorders but it can also be used for phototherapy of skin diseases. Inducible heme oxygenase-1 (HO-1) in response to a variety of stimuli, including UV exposure, is vital to maintain cell homeostasis. Heme oxygenase-2 (HO-2), another member of the heme oxygenase family, is constitutively expressed. In this review, we discuss how heme oxygenase (HO), a vital rate-limiting enzyme, participates in heme catabolism and cytoprotection. Phylogenetic analysis showed that there may exist a functional differentiation between HO-1 and HO-2 during evolution. Furthermore, depending on functions in immunomodulation and antioxidation, HO-1 participates in disease progression, especially in pathogenesis of skin diseases, such as vitiligo and psoriasis. To further investigate the particular role of HO-1 in diseases, we summarized the profile of the HO enzyme system and its related signaling pathways, such as Nrf2 and endoplasmic reticulum crucial signaling, both known to regulate HO-1 expression. Furthermore, we report on a C-terminal truncation of HO-1, which is generally considered as a signal molecule. Also, a newly identified alternative splice isoform of HO-1 not only provides us a novel perspective on comprehensive HO-1 alternative splicing but also offers us a basis to clarify the relationship between HO-1 transcripts and oxidative diseases. To conclude, the HO system is not only involved in heme catabolism but also involved in biological processes related to the pathogenesis of certain diseases, even though the mechanism of disease progression still remains sketchy. Further understanding the role of the HO system and its relationship to UV is helpful for revealing the HO-related signaling networks and the pathogenesis of many diseases.

## 1. Introduction

Heme oxygenase (HO) is an important rate-limiting enzyme and widely distributed in mammalian tissues. The HO system can degrade the heme into biliverdin (BV), free ferrous iron (Fe^2+^), and carbon monoxide (CO) [1]. These metabolic products participate in physiological processes including oxidative stress, inflammation, and apoptosis. The heme oxygenase occurs in two isoforms, HO-1 and HO-2 (gene names *HMOX1* and *HMOX2*). HO-1 is the inducible isoform that can be induced by a variety of environmental stimuli, such as UV radiation, heavy metal, lipopolysaccharide, heat shock, growth factors, hydrogen peroxide, phorbol esters, nitric oxide, inflammatory cytokines, endotoxins, hyperoxia, and hypoxia [2–5]. Hence, it is a general concept that HO-1 not only is an oxidative stress marker but also has some cytoprotective properties.

The study on the HO-1 transcriptional regulatory region shows the presence of regulatory sequences for the binding of various transcription factors such as AP-1, AP-2, NF-*κ*B, ATF4, Nrf2, Jun B, and HIF-1, which illustrates that HO-1 could also maintain cellular homeostasis [6, 7]. In contrast to *HMOX1*, only a few regulatory elements have been identified in the promoter region of *HMOX2*, such as a glucocorticoid response element (GRE). Indeed, corticosterone or dexamethasone treatment can increase the expression of HMOX2 [8].

Human HO-2 is constitutively expressed and plays a role in the production of CO in neuronal populations. In cerebral tissue, HO-2 is induced in response to cellular oxidative damage and NO sources whereas hypoxia could reduce its expression [9]. HO-2 is also a potential oxygen sensor through BK_Ca_ channel activity and hypoxic response in mammalian cells [10].

Despite the well-known role in heme catabolism, HO-1 participates in some disease progressions with properties of immunomodulatory and antioxidation, especially some skin diseases such as vitiligo and psoriasis.

UV as a common environmental factor for skin regulates HO-1 through a complicated signaling network. In this review, we explore the relationship between UVA and HO-1 and focus on Nrf2/Keap1-HO-1 and Eif2*α*-HO-1 signaling pathways, which are significant pathways in cellular antioxidation [11–13]. We elucidate the function of HO by introducing the transcripts of HO-1 and HO-2. Depending on different cell types, tissues, organs, and species, the HO system will generate various transcripts that may achieve distinctive functions. Different from HO-1, HO-2 renders several transcripts [14, 15]. A truncated form of HO-1 as a signal transducer localized to the nucleus was already introduced [16]. In previous studies, Bian et al. identified a novel isoform 14 kDa HO-1 form that might be related to tumor growth [17]. HO-1 is highly inducible following UVA irradiation in skin fibroblasts, with much lower levels in keratinocytes [18, 19]. Furthermore, we found that silencing of HO-2 in keratinocytes increases HO-1, which also further increases UVA-mediated HO-1 expression in HaCaT cells [20]. Although the vital function of the HO system in heme catabolism and maintenance of cell homeostasis has been well elucidated, recent new findings about the multifunctional role of the HO system in many skin diseases and UV irradiation are worthy to be reviewed in detail. Further understanding of the role of the HO system is helpful for revealing the pathogenesis of many diseases.

## 2. Heme Oxygenase System

### 2.1. The General Role of the Heme Oxygenase System in Heme Catabolism and Oxidative Stress

Heme oxygenase (HO) is the vital rate-limiting enzyme in heme catabolism and widely exists in mammalian tissues [21]. HO isoenzymes are located in the endoplasmic reticulum (ER) [22]. The enzyme can degrade the heme into biliverdin (BV), free ferrous iron (Fe^2+^), and carbon monoxide (CO) [1]. With the function of biliverdin reductase (BVR), biliverdin is converted to bilirubin (BR) and all the metabolic products of HO activity can participate in the physiological process including oxidative stress, inflammation, and apoptosis [23, 24]. The bile pigments biliverdin and bilirubin can scavenge ROS and nitrogen reactive species (NRS) through the recycling mechanism [25, 26]. It was noted that bilirubin can suppress the inflammatory response and decrease the cellular toxicity [27].

As a product of the HO enzymatic activity, CO can modulate the mitogen-activated protein kinase (MAPK) and p38*β* pathways to induce antiapoptotic, antiproliferative, and anti-inflammatory properties [28]. CO stabilizes the hypoxia-inducible factor 1*α* (HIF-1*α*), which plays a role in cytoprotection in macrophages. CO can inhibit cytochromes of the respiratory chain and NADPH oxidase (NOX), thus attributed to the reduction of ROS [29, 30]. Fe^2+^ is a product of the HO system and can be rapidly removed by ferritin to avoid the prooxidant capacity. With intracellular thiols, Fe^2+^ can form an iron-sulfur complex [3, 9, 31, 32]. The extreme hydrophobicity of heme can generate reactive oxygen species (ROS) and easily bind to the lipids leading to membrane lipid peroxidation. This can disrupt the membranes of several cellular organelles such as the endoplasmic reticulum (ER), nuclei, and cell membrane [33].

The HO system has the ability to keep the heme protein in balanced levels and protects cells from intracellular free heme damage [34]. Therefore, the cytoprotective role of the HO system is important in the biological process [35–37].

### 2.2. Homologous Alignment and Phylogenetic Analysis of HO System

**Figure 1:**
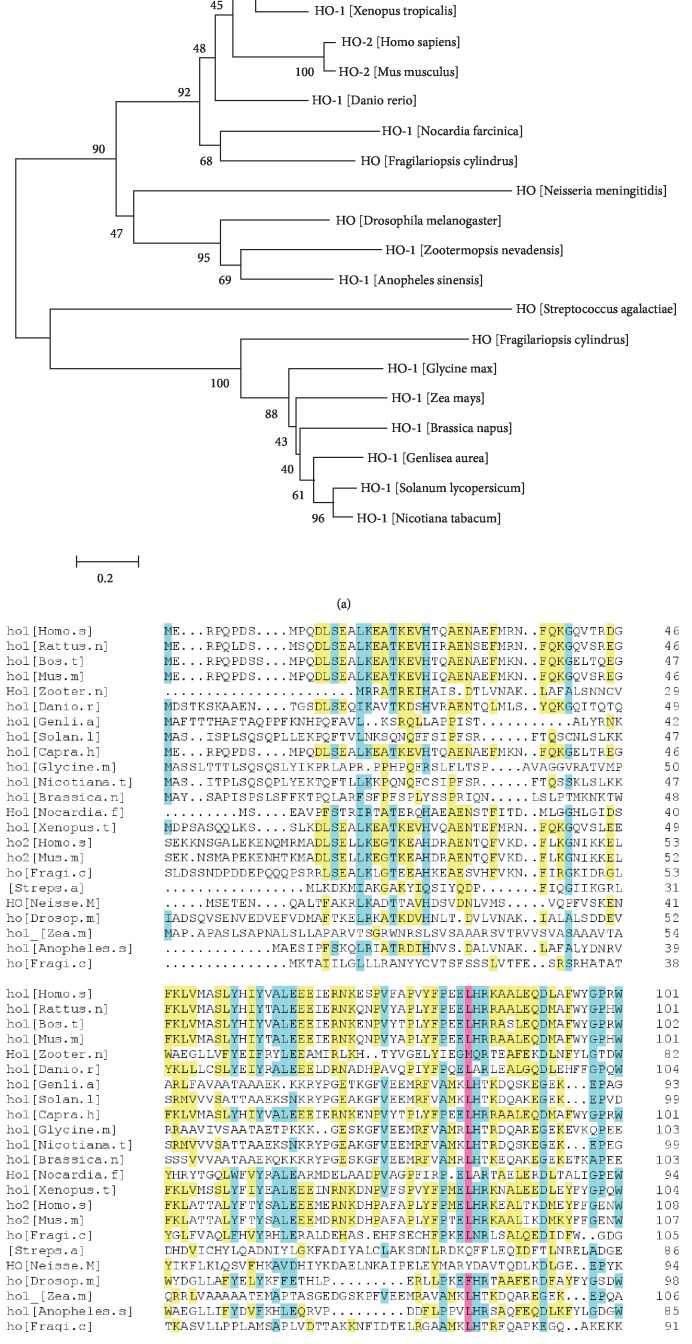
Homologous alignment and phylogenetic analysis of heme oxygenase and HO-like proteins. (a) Phylogenetic analysis of HO from different species. The amino acid sequences were downloaded from the NCBI website. Amino acid position is presented by a 0.2 bar. (b) Alignment of deduced HO proteins with other species.

Homologous alignment revealed that the *HMOX1* gene encodes 288 amino acids and *HMOX2* encodes 313 amino acids [38].  Figure 1 shows that *HMOX1* presents with 21.71% identity to *HMOX2* and *HMOX1* produced significant alignments with those from *Bos taurus* (41.37%), *Mus musculus* (40.51%), *Xenopus tropicalis* (29.57%), *Danio rerio* (21.71%), *Drosophila melanogaster* (9.23%), *Zootermopsis nevadensis* (7.18%), *Nicotiana tabacum* (4.44%), and *Zea mays* (4.10%). To analyze the evolutionary relationship of HO with the HO-like protein of other species, the neighbor-joining method was used to construct an HO phylogenetic tree. The results demonstrated that Hmox1 might have a closer relationship with *Bos taurus* and *Capra hircus*, while HMOX2 has a closer relationship with *Mus musculus* than other species (Figure 1).

### 2.3. Heme Oxygenase-1

The 32 kDa HO-1 protein belongs to a family of stress proteins as inducible isoform of *HO*, which is highly expressed in the liver, spleen, and bone marrow [39]. HO-1 can be induced by a variety of environmental stimuli, including UV radiation, heavy metals, lipopolysaccharides, heat shock, growth factors, hydrogen peroxide, phorbol esters, nitric oxide, inflammatory cytokines, endotoxins, hyperoxia, and hypoxia [2–5]. Due to its expression at low levels under basal conditions, but quickly upregulated, HO-1 has been recognized as a biomarker of oxidative stress. The human HO-1 gene is located on chromosome 22q12 and it contains 4 introns and 5 exons [40, 41]. It is generally cytoprotective, antiapoptotic, anti-inflammatory, and antioxidant [23].

We have explored the relationship between HO-1 and UVA. As an environmental factor, UVA irradiation releases heme from microsomes and generates reactive oxygen species (ROS), which could regulate HO-1 expression [42, 43].

Being a multifunctional molecule, HO-1 also participated in some skin diseases [44]. A recent report noted that HO-1 is a powerful immunomodulator, and elevated levels of HO-1 can eliminate inflammatory atopic dermatitis-like lesions in mice [45]. As a multifunctional protein, HO-1 can suppress dendritic cell maturation, T cell activation, and B cell infiltration [46]. In experimental models of ischemia/reperfusion, HO-1 has the ability to protect against cell death, thus making HO-1 a promising target in diverse disease phenotypes, such as myocardial infarction, sepsis, and stoke [47]. In endothelial cells (EC), HO-1 expression could protect EC from undergoing programmed cell death and the antiapoptotic property of HO-1 is mediated heme catabolism to the carbon monoxide (CO) [48]. The major molecular mechanism is when HO-1 inhibits the extrinsic and intrinsic apoptotic pathway, including elevated CO production wherein CO could inhibit P53 expression, decrease prooxidant levels, and increase bilirubin [49]. HO-1 could stimulate various types of cell proliferation and growth, and high levels of HO-1 expression occur in some tumors because of its antiapoptosis and antioxidation [50–52]. Depending on HO-1 which is related to the tumor growth, we are also provided a view that the HO-1 inhibitor could become a novel antitumor chemotherapy.

The function of HO-1 showed extreme similarities among the pathogenesis of vitiligo and psoriasis [53, 54]. In vitiligo, T cells mediated immune responses against melanocytes and against keratinocytes in psoriasis [55]. A previous study demonstrated that vitiligo melanocytes are equipped with the dysfunctional Nrf2-HO-1 antioxidant signaling pathway, as well as the aberrant expression of miRNAs [56–58]. Oxidative stress is considered as a contributing factor in T cell-mediated attack against melanocytes and therefore depigmentation of vitiligo skin; the dysfunctional Nrf2-HO-1 may contribute to pathogenesis of vitiligo. Furthermore, HO-1 expression has been associated with immunosuppressive effects, such as immunoregulatory function of Tregs [59]; the attenuated function of Tregs affecting progressive vitiligo has been confirmed [55, 60, 61]. In melanoma, HO-1 gene promoter mutations have been reported [57, 62]. Different from HO-1, HO-2 is constitutively expressed and has hardly been induced, but HO-2 still plays a vital role in heme homeostasis and antioxidation.

### 2.4. Heme Oxygenase-2

HO-2 is a 36 kDa protein that is encoded on human chromosome 16q12 [40]. HO-2 is mainly expressed in the brain, testis, spleen, neurons, and endothelial and glial cells [63]. In the brain, HO-2 is expressed in an abundant form, since HO-2 is constitutively expressed in neurons and is involved in antiapoptosis in the cortical and hippocampal [64, 65]. HO-2 acts in the production of CO in neuronal populations, and due to its high expression, in cerebral tissue, HO-2 can respond to cellular damage [9, 66]. Unlike HO-1, HO-2 is hardly inducible and can only be induced by NO donors, which is reduced by hypoxia [67, 68]. Owing to the deficient cysteine motifs in HO-1, HO-2 is a potential oxygen sensor through the BK_Ca_ channel activity and hypoxic response in mammalian cells [10]. In contrast to HO-1, HO-2 is mainly constitutively expressed and a few regulatory elements have been identified in the promoter region of HO-2 [9], such as a glucocorticoid response element (GRE) [8]. The expression of HO-2 can be induced under a few conditions. It is upregulated by adrenal glucocorticoids; in endothelial cells, estrogen also upregulates HO-2 [8]. A previous study noted that adrenal glucocorticoids can also modulate the HO-2 expression [8]. In cerebral and smooth muscle cells, HO-2 is also activated by glutamate and increased CO production. As an enzyme, HO-2 activity can be affected by posttranslational modifications; it can also be regulated by the presence of NO and ROS [9, 69]. Basal levels of HO-2 have the ability to maintain heme homeostasis; meanwhile, it can protect against cellular oxidative stress as well [70]. In contrast to HO-2, there are still some publications that reported that HO-1 is a multifunctional protein involved in some vital biological processes and further investigating its transcriptional regulation has become a matter of significance.

## 3. Ultraviolet Radiation and HO System

Ultraviolet (UV) light is electromagnetic radiation with wavelengths in the range of 200-400 nm. Based on the wavelength of UV light, it can be divided into three parts, UVA (320-400 nm), UVB (280-320 nm), and UVC (lower than 280 nm) [71, 72]. In general, the solar radiation is an environmental factor, which can trigger some skin diseases, such as polymorphic solar eruption (PMLE), photoaging, and skin cancer. Melanoma, squamous cell carcinoma (SCC), and basal cell carcinoma (BCC) are the three main types of skin cancer, and UV radiation (UVR) is the major risk factor for the occurrence of skin cancers [73]. Melanin, produced in melanocytes, plays a critical role in protecting against UV-mediated mutagenesis. However, a recent study observed a decrease in the risk of melanoma and nonmelanoma skin cancer in vitiligo subject with the absence of melanin in vitiligo skin, which may be explained by the inverse relationship between the risk of vitiligo and skin cancers in the RALY-EIF252-ASIPAHCY-ITCH, IRF4, TYR, and MC1R genes [74–77]. UVR could disrupt skin keratinocytes, which cause inflammatory disorders. However, UV radiation exhibited both beneficial and detrimental effects. Ultraviolet radiation, including narrowband UVB (311-313 nm), broadband UVB (290-320 nm), and UVA-1 (340-400 nm), was employed as phototherapy for several chronic inflammatory skin diseases, including atopic dermatitis, vitiligo, pruritus, cutaneous mastocytosis, and psoriasis [78–80].

Long-time exposure to UVA radiation can accumulate reactive oxygen species (ROS), which leads to cellular oxidative stress and activates antioxidation pathways [81]. High doses of UVA (>300 J) can cause DNA damage in either direct or indirect ways related to pathogenesis [82]. Our lab has shown that different wavelengths of UV can activate specific signal pathways [13, 83–85]. As the long wavelength UVA radiation mainly exists in the living environment, it has attracted our attention to UVA radiation research.

HO-1, which belongs to the heme oxygenase family, can be upregulated by low and medium doses of UVA irradiation; the induction of HO-1 contributes to cellular redox homeostasis. We have explored the relationship between HO-1 and UVA. As an environmental factor, UVA irradiation releases heme from microsomal and generates reactive oxygen species (ROS), which could regulate HO-1 expression [42, 43]. Both UVA and UVB can induce HO-1 expression, though much higher levels of induction were found for UVA irradiation. When UVA induction of Nrf2 and HO-1 is abolished in skin cells, they are more sensitive to oxidative stress, such as UVA and H_2_O_2_, indicating that the Nrf2/HO-1 system has a protective role in skin cells [86].

## 4. UV-Related Signal Pathways and Transcription Involved in Regulation of HO-1

### 4.1. Transcription Regulation of HO-1

In humans, *HMOX1* transcription is involved in a variety of signal transduction pathways that activate different transcription factors. HO-1 can be upregulated by various inducers, and the transcriptional regulation is essential to explore the relationship between UVA and HO-1. It is well known that UVA is an oxidative agent, so we mainly focused on the molecular mechanism of UVA which actives the antioxidant signal pathways which affect the transactivation of *HMOX1* and other antioxidant genes [87–89]. Previously, transcription factor binding sites have been identified in the HO-1 promoter region, such as AP-1, AP-2, NF-*κ*B, ATF4, Nrf-2, Jun B, and HIF-1, which are associated with the immediate response to tissue injury, inflammatory, and oxidation stress [6, 90].

AP-1 binding sites have been identified, which suggest that a contribution of Jun/Fos transcription factor family induces HO-1 gene transcription by multiple agents [91]. AP-2 and NF-*κ*B binding sites may be implicated as HO-1 in response to tissue injury, oxidation stress, cell growth control, and differentiation processes. As for the NF-*κ*B transcription factor, it is involved in many cell type challenges and pathogenic stimuli, including virus, bacterial, stress, and inflammatory cytokines. HIF-1 is a factor that is related to hypoxia, and ATF4 is an activating transcription factor that can upregulate some genes [12, 92, 93].

### 4.2. Nrf2/Keap1-HO-1 Signaling

Nrf2 (nuclear factor erythroid-derived 2 related factor 2) belongs to the basic leucine zipper family of transcription factors and is responsible for the regulation of cellular redox balance and antioxidation [94]. The antioxidant response element (ARE) is attributed to a consensus binding sequence, identified in HMOX1, thioredoxin reductase 1 (Txnrd1), and series of antioxidative genes [95]. Antioxidation genes could be induced in response to environmental stimuli, such as UV. The procedural activation of cascade affects the status of the cells and provides protection against cellular oxidative stress [96]. Apart from Nrf2, some factors like Nrf1 and Nrf3 as well as transcriptional repressors Bach1 and Bach2 are also members of the bZIP family of transcription factors [97]. Keap1 is a cysteine-rich protein, serving as an adaptor protein for the Cul3-dependent E3 ubiquitin ligase complex [98–100].

Under oxidative stress, including UV irradiation, Nrf2 is separated from Keap1 and translocates to the cell nucleus [95]. Nrf2 combines with small Maf proteins (sMaf) and CBP (CREB-binding protein) and then binds to the antioxidant responsive elements (ARE) in the promoters of target genes [101]. However, Nrf2 can be degraded in the nucleus via the *β*-TrCP-GSK3*β* axis or it may translocate back to the cytoplasm and is degraded by Keap1 [102]. Under normal conditions, Keap1 promotes ubiquitination and degradation of Nrf2 and Nrf2 exhibiting a short nearly 20 min half-life, which keeps the low level of Nrf2 to maintain cellular homoeostasis [103]. Keap1, as a thiol-rich protein, contains cysteine residues; the Cys273 and Cys288 are important for Keap1 to regulate Nrf2 under oxidation stress conditions and Cys151 is vital to active Keap1 under cellular stress conditions [99, 104, 105]. It was found that silencing of Keap1 increases the expression of HO-1 by several fold [103].

Therefore, the Nrf2/Keap1-HO-1 pathway is an indispensable route to minimize oxidative stress. Nrf2 is an essential factor through binding to the Maf recognition element (MARE) thereby activating the antioxidant responsive element (ARE), which participates in oxidative stress response [106]. We conclude that Keap1 acts as a sensor in response to oxidative stress and leads to translocation of activated Nrf2 which in turn regulates transcription of a series of antioxidant genes, including HO-1, so that the Nrf2/Keap1-HO-1 signaling pathway is sensitive to oxidative stress.

### 4.3. Bach1/HO-1 Signaling

Both Bach1 and Bach2 consist of the BTB and CNC homology family, as a transcription factor that belongs to the basic region-leucine zipper factor family (bZIP) [97, 107]. In general, Bach1 and Bach2 form heterodimers with sMaf proteins and bind to the MARE to become transcription repressors [108, 109]. The BTB domain is required for protein-protein interactions and the bZIP domain possesses the nuclear localization signal [107, 110]. Bach2 has the ability to bind a TPA (12 O-tetra decanoylphorbol-13-acetate) response element (TRE) in its promoter region (5′UTR). Except for TRE, Bach2 can also bind to MARE (MAF response element) and ARE (antioxidant response element) in complex with the MAF protein, which results in repressed transcription. Depending on the same consensus sequence (TGAG/CTCA), TRE, MARE, and ARE elements can be bound by Maf family proteins [111]. Bach1 as a competitive binder to the ARE motifs leads to exclusion of Nrf2. As a repressive transcription factor, Bach1 regulates gene induction by release from enhancer elements. Otherwise, Bach1 plays a vital role in the Nrf2/Keap1-HO-1 pathway. Silencing of Bach1 increases HO-1 mRNA and protein dramatically, and the strong suppression of HO-1 activation is primarily mediated by Bach1 in HaCaT cells [43]. The phenomenon also illustrates that competitive binding of Bach1 to ARE motifs ensures that ARE motifs are not overstimulated by oxidation so that this mechanism probably maintains the heme balance by tightly regulating HO-1 expression.

### 4.4. PERK-HO-1 Signaling

In addition to the Nrf2-HO-1 pathway, there is another important signal pathway that can regulate the expression of HO-1. The PERK-ATF4-HO-1 pathway, which belongs to the cellular homeostatic pathways, can be activated by integrated stress response (ISR) [12, 112]. UVA irradiation leads to oxidative stress and generation of ROS, and all these stressors may trigger disruptions of endoplasmic reticulum (ER) homeostasis, thereby causing ER stress [113, 114]. PERK belongs to the transmembrane ER receptor with a serine/threonine cytoplasmic domain; activated protein kinase RNA-like endoplasmic reticulum kinase (PERK) makes the eukaryotic initiation factor 2 alpha (eIF2*α*) phosphorylation, especially for Ser51 phosphorylation of eIF2*α*, and affects the repression of global protein synthesis and preferential translation of selected genes [114, 115]. In the mouse epidermal cell, Xue et al. found that UVA irradiation could activate eIF2*α* phosphorylation and Nrf2-HO-1 signaling and that modulated eIF2*α* phosphorylation status could change the Nrf2-HO-1 pathway [116].

**Figure 2:**
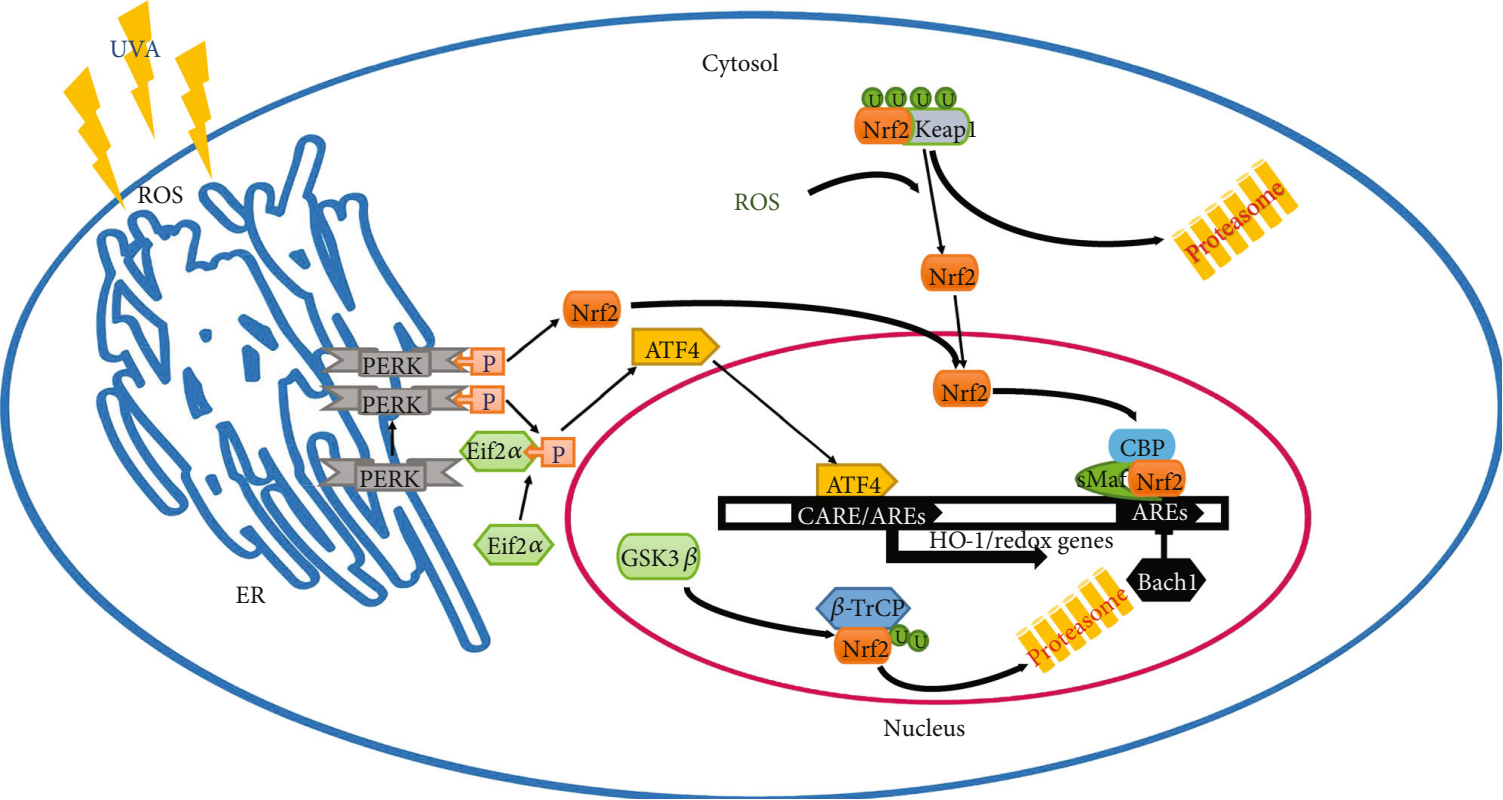
The mechanism of UVA-modulated HO-1 regulation through the PERK and Nrf2 signal pathway. U: ubiquitination; P: phosphorylation; ROS: reactive oxygen species; ARE: antioxidant response elements; CARE: C/EBP-ATF regulatory element; HO-1: heme oxygenase-1; ER: endoplasmic reticulum; Maf: small Maf protein; Bach1: transcription repressor; Nrf2: nuclear factor 2-related erythroid factor-2; CBP: CREB-binding protein.

Increasing eIF2*α* phosphorylation enhanced expression of activating transcription factor 4 (ATF4); ATF4 is a bZIP transcription factor that can be upregulated by multiple effectors that determine cell fate [117, 118]. Since ATF4 is downstream of PERK, it could participate in the metastatic cascade and is also critical for the regulation of autophagy [12]. ATF4 transcriptionally regulates several antioxidant genes in response to oxidative stress, including HO-1 and superoxide dismutase 2 (SOD2) [119, 120]. In general, ATF4 regulates the expression of genes by mainly binding to C/EBP-ATF regulatory elements (CARE) in the gene promoter region; however, latest studies of the HO-1 promoter have shown that ATF4 binds to unique ARE sites in the HO-1 promoter and interacts with Nrf2 to upregulate expression following matrix detachment [12, 121]. However, PERK directly phosphorylates Nrf2 to activate a cascade of antioxidant signaling. Nrf2 is also widely regarded as the primary transcriptional inducer of HO-1, which implies a cooperative activity of ATF4 and Nrf2 that may regulate the transcription of HO-1 [11, 12]. It also reveals that the intersection node between PERK-eIF2*α* and PERK-Nrf2 signaling toward regulating the transcription of HO-1 suggests that PERK could potentially be a therapeutic target for disease [122–124]. For further studies, various pathways can be activated in association with distinct wavelengths of UV, especially PERK-eIF2*α* signaling. It is known that ER stress signaling in response to unfolded protein stress (UPR) and based on diverse degrees of UPR could determine cell fate through the ER stress pathway. However, PERK can phosphorylate not only eIF2*α* but also Nrf2 [11]. Increasing eIF2*α* phosphorylation enhanced ATF4 expression and ATF4 could also regulate HO-1 expression, as shown in Figure 2. Zong et al. found that 60Co*γ* radiation induces ATF4 mRNA and protein expression in a dose- and time-dependent manner in AHH1 lymphoblast cells. Following 60Co*γ* radiation, ATF4 expression was increased in murine spleen cells, endothelial cells, and liver LO2 cells [125]. ATF4 is sensitive to ionizing radiation, which further confirms that HO-1 in response to diverse radiation modes may be related to ATF4 as an inducer. Therefore, we hypothesized that there may be a cross-talk relation between ATF4 and Nrf2 signaling through phosphorylation cascades (Figure 2). The phosphorylation status probably demonstrates the dose equivalent of radiation and also provides a new way to explore the principles of biological processes in response to different wavelength radiation modes.

### 4.5. Bioinformatics Analyzation of the HO-1 5′UTR Region

In order to further illustrate the mechanism of HO-1 regulatory relation, MatInspector was performed to predict the 5′-flanking region and some cis-regulatory elements (CREs) that were detected in the HO-1 5′-untranslated region. Although there are still some diverse mutations that exist on the different types of the human HO-1 promoter region, it is sufficient information to illustrate the potential regulatory relationship.  Table 1 shows the AARE binding factors that were found. An ATF4 binding site means that either ATF4 or a heterodimer of CEBP epsilon and ATF4 could regulate HO-1 expression. Activator protein 1 could be induced in response to stimuli that have been reported. Estrogen-related receptor alpha binding site showed that estrogen could affect HO-1 and HO-1 expression suggesting that HO-1 levels may be different in male vs. female organisms. In addition, a binding site for the leucine zipper protein NF-E2 was predicted. A binding site for the C/EBP homologous protein (CHOP) could mean that HO-1 may be involved in the apoptosis process. Heat shock factor 1 showed that heat temperature difference may affect HO-1 expression. The hypoxia-inducible factor, bHLH/PAS protein family, is related to oxygen deficit. Nuclear factor kappa B (p50, p65) may be involved in inflammatory response. Signal transducer and activator of transcriptions 1, 3, 5, and 6 were likely related to signal transmission and proliferation. Besides, there are still some attractive binding sites that were found by predication, such as autoimmune regulatory element binding factor, nuclear factor Y binding factor, calcium-response factor, tumor suppressor p53, and tumor protein p63, which require further investigations to demonstrate that these factors are relevant for HO-1 regulation.

## 5. The Effects of HO Transcripts and Truncated HO-1

### 5.1. Transcripts of HO

As opposed to HO-1, HO-2 possesses some transcripts [15, 126]. Depending on different cell types, tissues, organs, and species, it generates various transcripts that have distinct functions. Different sizes of HO-2 transcripts have been identified; most of them are associated with tissue- and development-specific regulation [14]. However, in other species, HO-1 and HO-2 exert a similar mechanism. Various HO-2 transcripts can be generated by alternative splicing, alternative usage of polyadenylation sites, stage-specific exon utilization, or transcriptional site initiation [127]. The promoter region is important for the transcript formation [128, 129]. There also exists evidence that genetic variations of HMOX1 impact on the physiological function [14], especially for the single nucleotide polymorphism (SNP) and a microsatellite GT-dinucleotide repeat in the promoter region that is related to incidence and progression of disease [6, 130]. These polymorphisms may be a potential component of the pathogenesis through HMOX-1 transcription or translation regulation. The length of the polymorphism is also associated with susceptibility to many diseases such as cardiovascular disease, peripheral artery diseases, lung adenocarcinoma, and Parkinson's disease [131–136]. Moreover, common polymorphisms usually can affect alternative splicing [137]. A recent report by Bian et al. identified an alternative splice isoform of 14 kDa HO-1, which may be involved in tumor growth and telomere modulation [17]. The alternative splice isoform of HO-1 has been found, which helps to clarify its potential function in diseases and provides some meaningful data.

### 5.2. Truncated HO-1

In mouse 3T3 cells, a 28 kDa HO-1 band was induced under hypoxic exposure; the 28 kDa HO-1 was primarily localized to the nucleus and known as nuclear proteins. This isoform of HO-1 missing 52 amino acids from the C terminus was found to be enzymatically inactive [16]. Hori et al. showed that an enzymatically inactive form of HO-1 was also able to protect against oxidation damage; it can bind to heme but cannot degrade it into biliverdin [138]. In addition, Kassovska-Bratinova et al. used mass spectroscopy to identify a 27 kDa nuclear form of HO-1 that lacks the C terminus [139].

The truncation of the C terminus of human HO-1 by 23 amino acids maintains enzyme activity, but further truncation by 56-68 amino acids reduces HO activity [140]. The C-terminal truncation of HO-1 does not alter the heme catalytic pocket [16]. Meanwhile, the truncated HO-1 modulates stabilization and nuclear accumulation of Nrf2, so the truncated HO-1 protein may play a role in cellular signaling through migration to the nucleus or affect nuclear transcription [141].

There are several examples of cytoplasmic enzymes serving functions in the nucleus; we are also interested in the procession of HO-1 translocation to the nucleus. In general, nuclear localization sequences (NLS) are essential for the majority of proteins that migrate to the nucleus. So far, no NLS has been identified in HO-1; however, HO-1 has a nuclear export sequence (NES). The oxidative modification can modify the function of an NES [142]. In most instances, CRM1 binds with RanGTP to form a complex to allow the nuclear pore through to the cytoplasm [143].

HO-1 may bind to the CRM1 complex for nuclear import rather than for nuclear export [144]. This suggests that CRM1 may shuttle across the nuclear pore and that truncated HO-1 may participate in intercellular signaling [16, 20]. According to the HO transcripts and truncated HO-1, we found that the HO system may play a vital role in cellular homeostasis, which transforms into different transcriptional profiles and performs diverse functions.

## 6. Conclusion

HO proteins are vital rate-limiting enzymes, which participate in heme catabolism and protect against cellular oxidative stress. HO-1 can be induced by UV irradiation [145], and UV regulates several pathways involving phosphorylation of eIF2*α*, phosphatidylinositol- (PI-) 3 kinase, mitogen-activated protein kinases (MAPKs), ATM, and ATR [13, 146–148]. A complex signaling network between HO-1 and UV irradiation has recently been revealed, including Nrf2-HO-1 signaling, eIF2*α*-ATF4-HO-1 signaling and Bach1/HO-1. The phosphorylation of eIF2*α* could induce HO-1 expression. Moreover, Nrf2/Keap1-HO-1 signaling is another crucial antioxidative signaling pathway that is activated in response to UV exposure. PERK not only phosphorylated Nrf2 but also phosphorylated eIF2*α*, suggesting that there may exist a relation between Nrf2 and ER stress signaling. Furthermore, We also introduced a new form of truncated HO-1 which is revealed to be related to tumor growth and telomere modulation. Associated with immunomodulation and antioxidation, HO-1 plays a crucial role in pathology. Taken together, this review describes the character of the HO enzyme system, and its relationship to UV is helpful for revealing the HO-related signaling networks and the pathogenesis of many diseases, which also might provide new insights into potential therapeutic applications, i.e., by manipulating potential genetic targets.

## Figures and Tables

**Table 1:** The predication of HO-1 promoter region.

Gene	Detail	Matrix name	Start	End	Ration	Strand	Sequence
AARE binding factors	ATF4 binding site	V$AARE.01	361	369	0.953	(−)	gTTTCacca
AARE binding factors	ATF4 binding site	V$AARE.01	2098	2106	0.953	(+)	gTTTCacca
Autoimmune regulatory element binding factor	Autoimmune regulator	V$AIRE.01	1834	1848	0.785	(+)	ccTTATttatggggt
AP1, activating protein 1	Basic leucine zipper transcription factor, ATF-like	V$BATF.01	807	819	0.977	(−)	cattgaCTCAagg
AP1, activating protein 1	Activator protein 1	V$AP1.02	1208	1220	0.892	(+)	atctGAGTgagcc
AP1, activating protein 1	Transcription factor Jun B	V$JUNB.01	807	819	0.977	(+)	ccttgaGTCAatg
MAF- and AP1-related factors	Heterodimers with small Maf proteins	V$MARE.03	669	693	0.92	(+)	gctctgGCTGaatcatgaaagggga
MAF- and AP1-related factors	Bach2 bound TRE	V$BACH2.01	802	826	0.951	(+)	catcccctTGAGtcaatgctgttat
MAF- and AP1-related factors	Leucine zipper protein NF-E2 (nuclear factor, erythroid-derived)	V$NFE2.02	2530	2554	0.805	(+)	ggggctggcgaGTCActgacccgcc
MAF- and AP1-related factors	NF-E2 p45	V$NFE2.01	2707	2731	1	(+)	gattttgCTGAgtcaccagtgcctc
Activator protein 2	Transcription factor AP-2, beta	V$TCFAP2B.01	1173	1187	0.891	(−)	ggaGCCCctgggccc
Heterodimers between bZIP family members	Heterodimer of CEBP epsilon and ATF4	V$CEBPE_ATF4.02	1678	1690	0.89	(−)	aattatGCAAgct
Heterodimers between bZIP family members	Heterodimer of CEBP epsilon and ATF4	V$CEBPE_ATF4.02	1960	1972	0.869	(+)	agtggtGCAAtct
Heterodimers between bZIP family members	Heterodimer of CEBP epsilon and ATF4	V$CEBPE_ATF4.02	2907	2919	0.883	(+)	aatgttGCAAtcc
CCAAT binding factors	Nuclear factor Y (Y-box binding factor)	V$NFY.04	2192	2206	0.922	(−)	tgggCCAAttgtggt
CCAAT binding factors	Nuclear factor Y (Y-box binding factor)	V$NFY.01	3692	3706	0.91	(+)	ctttCCAAtgggggg
CCAAT binding factors	Nuclear factor Y (Y-box binding factor)	V$NFY.04	1663	1677	0.948	(+)	tgatCCAAttagact
Calcium-response elements	Calcium-response factor	V$CARF.01	752	762	0.936	(−)	acagtGAGGct
Calcium-response elements	Calcium-response factor	V$CARF.01	2437	2447	0.911	(−)	agagtGAGGag
Cell cycle regulators: cell cycle-dependent element	Cell cycle-dependent element, CDF-1 binding site	V$CDE.01	2609	2621	0.937	(+)	gagtCGCGatttc
C/EBP homologous protein (CHOP)	Heterodimers of CHOP and C/EBPalpha	V$CHOP.02	1978	1990	0.974	(+)	cacTGCAacctcc
Cell cycle regulators: cell cycle homology element	CDE/CHR tandem elements regulate cell cycle-dependent repression	V$CHR.01	3922	3934	0.97	(+)	tagtTTGAatcct
C/EBP homologous protein (CHOP)	Heterodimers of CHOP and C/EBPalpha	V$CHOP.01	2909	2921	0.923	(+)	tgttGCAAtccac
cAMP-responsive element binding proteins	c-Jun/ATF2 heterodimers	V$CJUN_ATF2.01	1362	1382	0.998	(+)	ccttgcTGACttcaaggagct
cAMP-responsive element binding proteins	Activating transcription factor 1	V$ATF1.02	2643	2663	0.918	(−)	caaacaTGACgcagcagaaat
cAMP-responsive element binding proteins	X-box-binding protein 1	V$XBP1.01	6597	6617	0.93	(−)	ggcgggtcACGTgggccaggc
CTCF and BORIS gene family	Insulator protein CTCF (CCCTC-binding factor)	V$CTCF.05	3782	3808	0.834	(−)	attcagcgaccctAGAGggtaaaactc
E2F-myc activator/cell cycle regulator	E2F transcription factor 4, p107/p130-binding protein	V$E2F4.01	2542	2558	0.969	(−)	gggggGCGGgtcagtga
Estrogen-related receptors	Estrogen-related receptor alpha	V$ESRRA.01	4197	4219	0.9	(−)	aatagatgcttcAAGGtctcttt
Estrogen-related receptors	Estrogen-related receptor alpha, homodimer DR5 binding site	V$ESRRA.05	1331	1353	0.829	(+)	taagggcataagAAGGtgaatct
Fork head domain factors	Alternative splicing variant of FOXP1, activated in ESCs	V$FOXP1_ES.01	1466	1482	1	(−)	gctgtaaAACAgattct
Fork head domain factors	Hepatic nuclear factor 3 beta (FOXA2)	V$HNF3B.03	4490	4506	0.886	(−)	cttttgTAAAgaagtgt
Glucocorticoid responsive and related elements	Progesterone receptor binding site, IR3 sites	V$pre.01	3545	3563	0.892	(−)	caggccagagtTGTTctgg
Glucocorticoid responsive and related elements	Androgene receptor binding site, IR3 sites	V$are.02	5282	5300	0.903	(+)	ggacagcgtcttGTTCtgt
Human acute myelogenous leukemia factors	Runt-related transcription factor 2/CBFA1	V$AML3.01	2567	2581	0.91	(−)	agaaGTGGtttgggc
Heat shock factors	Heat shock factor 2	V$HSF2.02	195	219	0.961	(−)	tttcaaatgtccAGAAaagtcgcaa
Heat shock factors	Heat shock factor 1	V$HSF1.02	481	505	0.753	(+)	tgcaatgagcCGAGatagtgccatt
Hypoxia-inducible factor, bHLH/PAS protein family	Hypoxia-inducible factor, bHLH/PAS protein family	V$HIF1.02	6599	6615	0.964	(+)	ctggcccaCGTGacccg
Hypoxia-inducible factor, bHLH/PAS protein family	Aryl hydrocarbon receptor nuclear translocator-like, homodimer	V$ARNTL.01	6600	6616	1	(−)	gcgggtcaCGTGggcca
Myc-associated zinc fingers	Myc-associated zinc finger protein (MAZ)	V$MAZ.01	2661	2673	0.951	(+)	ttggGAGGgggga
Myc-associated zinc fingers	MYC-associated zinc finger transcription factor	V$MAZR.01	2663	2675	0.885	(+)	gggaggGGGGact
Cellular and viral myb-like transcriptional regulators	v-Myb, AMV v-myb	V$VMYB.04	3596	3616	0.89	(+)	gggctgttctAACGaagtctg
NGFI-B response elements	Nuclear hormone receptor NUR77 (NR4A1)	V$NUR77.01	5088	5102	0.93	(−)	gacaaaaGTCAggtt
NeuroD, beta2, HLH domain	Neuronal differentiation 1	V$NEUROD1.02	5648	5662	0.929	(+)	gaatCATCtggtcca
Nuclear factor kappa B/c-rel	NF-kappaB (p50)	V$NFKAPPAB50.01	1629	1643	0.882	(−)	gagGGGAttaccaag
Nuclear factor kappa B/c-rel	NF-kappaB	V$NFKAPPAB.01	2396	2410	0.944	(−)	agGGGAcgtccccag
Nuclear factor kappa B/c-rel	NF-kappaB (p65)	V$NFKAPPAB65.01	4464	4478	0.992	(+)	tctggaatTTCCtta
“Negative” glucocorticoid response elements	Repressive binding sites for glucocorticoid receptor (IR2)	V$IR2_NGRE.01	1182	1196	0.803	(+)	ggCTCCtagggcagc
Nuclear receptor subfamily 2 factors	DR1 binding sites for TR2 homodimers or TR2/TR4 heterodimers	V$TR2_TR4.01	612	636	0.841	(−)	tgcagtagggcgggGGTCactaaca
Nuclear receptor subfamily 2 factors	TR4 homodimer, DR1 site	V$TR4.02	2740	2764	0.782	(+)	ctctttAGGTgggaggtgaaagggc
OVO homolog-like transcription factors	Zinc finger transcription factor OVO homolog-like 1	V$OVOL1.01	3600	3614	0.836	(−)	gacttcGTTAgaaca
p53 tumor suppressor	Tumor suppressor p53	V$P53.08	399	423	0.889	(+)	ctgggcatggtggtgCATGcctgta
p53 tumor suppressor	Tumor protein p63	V$TP63.02	5402	5426	0.933	(+)	acaggcatgtgccacCATGcccagc
Peroxisome proliferator-activated receptor	Peroxisome proliferator-activated receptor gamma, DR1 sites	V$PPARG.03	540	562	0.852	(+)	tctaaaaaaaaaAAAGgtaaata
SOX/SRY-sex/testis	SRY-box containing gene 3	V$SOX3.01	1765	1787	0.979	(−)	aacagaCAAAagatgaacattcc
Sterol regulatory element binding proteins	Sterol regulatory element binding protein	V$SREBP.03	3083	3097	0.948	(−)	tgaTCACctgaggtc
Signal transducer and activator of transcription	Signal transducer and activator of transcription 3	V$STAT3.02	4259	4277	0.966	(−)	ccagTTCCtggaatagtgc
Signal transducer and activator of transcription	STAT5: signal transducer and activator of transcription 5	V$STAT5.01	5105	5123	0.927	(−)	aaggTTCCccgaaaaccaa
Signal transducer and activator of transcription	STAT6: signal transducer and activator of transcription 6	V$STAT6.01	5117	5135	0.92	(−)	cttcTTCCtctgaaggttc
Signal transducer and activator of transcription	Signal transducer and activator of transcription 1	V$STAT1.01	5810	5828	0.793	(+)	aatgtgcctGGAAgagtgt
TGF-beta induced apoptosis proteins	Cysteine-serine-rich nuclear protein 1	V$CSRNP1.01	2165	2171	1	(+)	AGAGtgc
TCF11 transcription factor	TCF11/LCR-F1/Nrf1 homodimers	V$TCF11.01	3967	3973	1	(−)	GTCAttt
Activator/repressor binding to transcription initiation site	Transcription factor yin yang 2	V$YY2.01	3156	3178	0.981	(+)	catcctCCATcttcaaagctagc
Activator/repressor binding to transcription initiation site	Transcription factor yin yang 2	V$YY2.02	3794	3816	0.853	(−)	gtctgtCCATtcagcgaccctag
Members of ZIC family, zinc finger protein of the cerebellum	Zinc finger protein of the cerebellum (Zic3)	V$ZIC3.03	2978	2992	0.918	(+)	gcctcCAGCaggaga
